# Advances and challenges in automated malaria diagnosis using digital microscopy imaging with artificial intelligence tools: A review

**DOI:** 10.3389/fmicb.2022.1006659

**Published:** 2022-11-15

**Authors:** Carles Rubio Maturana, Allisson Dantas de Oliveira, Sergi Nadal, Besim Bilalli, Francesc Zarzuela Serrat, Mateu Espasa Soley, Elena Sulleiro Igual, Mercedes Bosch, Anna Veiga Lluch, Alberto Abelló, Daniel López-Codina, Tomàs Pumarola Suñé, Elisa Sayrol Clols, Joan Joseph-Munné

**Affiliations:** ^1^Microbiology Department, Vall d’Hebron Research Institute, Vall d’Hebron Hospital Campus, Barcelona, Spain; ^2^Universitat Autònoma de Barcelona (UAB), Barcelona, Spain; ^3^Computational Biology and Complex Systems Group, Physics Department, Universitat Politècnica de Catalunya (UPC), Castelldefels, Spain; ^4^Data Base Technologies and Information Group, Engineering Services and Information Systems Department, Universitat Politècnica de Catalunya (UPC), Barcelona, Spain; ^5^Clinical Laboratories, Microbiology Department, Hospital Universitari Parc Taulí, Sabadell, Spain; ^6^CIBERINFEC, ISCIII- CIBER de Enfermedades Infecciosas, Instituto de Salud Carlos III, Madrid, Spain; ^7^Probitas Foundation, Barcelona, Spain; ^8^Image Processing Group, Telecommunications and Signal Theory Group, Universitat Politècnica de Catalunya (UPC), Barcelona, Spain

**Keywords:** malaria diagnosis, digital imaging techniques, deep learning, artificial intelligence, microscopic examination, smartphone application, malaria

## Abstract

Malaria is an infectious disease caused by parasites of the genus *Plasmodium* spp. It is transmitted to humans by the bite of an infected female *Anopheles* mosquito. It is the most common disease in resource-poor settings, with 241 million malaria cases reported in 2020 according to the World Health Organization. Optical microscopy examination of blood smears is the gold standard technique for malaria diagnosis; however, it is a time-consuming method and a well-trained microscopist is needed to perform the microbiological diagnosis. New techniques based on digital imaging analysis by deep learning and artificial intelligence methods are a challenging alternative tool for the diagnosis of infectious diseases. In particular, systems based on Convolutional Neural Networks for image detection of the malaria parasites emulate the microscopy visualization of an expert. Microscope automation provides a fast and low-cost diagnosis, requiring less supervision. Smartphones are a suitable option for microscopic diagnosis, allowing image capture and software identification of parasites. In addition, image analysis techniques could be a fast and optimal solution for the diagnosis of malaria, tuberculosis, or Neglected Tropical Diseases in endemic areas with low resources. The implementation of automated diagnosis by using smartphone applications and new digital imaging technologies in low-income areas is a challenge to achieve. Moreover, automating the movement of the microscope slide and image autofocusing of the samples by hardware implementation would systemize the procedure. These new diagnostic tools would join the global effort to fight against pandemic malaria and other infectious and poverty-related diseases.

## Introduction

Malaria is one of the most common infectious diseases worldwide. It is caused by *Plasmodium* parasites and transmitted to humans by the bite of an infected female mosquito of the *Anopheles* genus. Over 241 million malaria cases were estimated in 2020, an increase from the 227 million of 2019 according to the World Health Organization (WHO) (*World Malaria Report* WHO 2021). Malaria is endemic in 85 countries and caused 627,000 deaths in 2020. Africa is the most affected continent with 95% of all malaria cases reported and 96% of all deaths (Talapko et al., 2019; *World Malaria Report* WHO 2021). Low-income countries with non-accessible healthcare resources are the most affected regions and malaria-related mortality has a high correlation with poverty rates (Ren, 2019). Socioeconomic data were collected in several studies to demonstrate the aforementioned correlation, describing the global health situation of malaria in low-income countries (Ricci, 2012; Konishi et al., 2016). An early diagnosis, suitable treatment, and prevention strategies such as vaccination or mosquito net control are crucial to fighting the infection. Due to its high global health impact, this infectious disease is still a global issue. In addition, the COVID-19 pandemic has increased the number of malaria deaths and cases from previous years, due to the high impact of this pandemic on the administration of healthcare resources worldwide (Heuschen et al., 2021).

**Figure 1:**
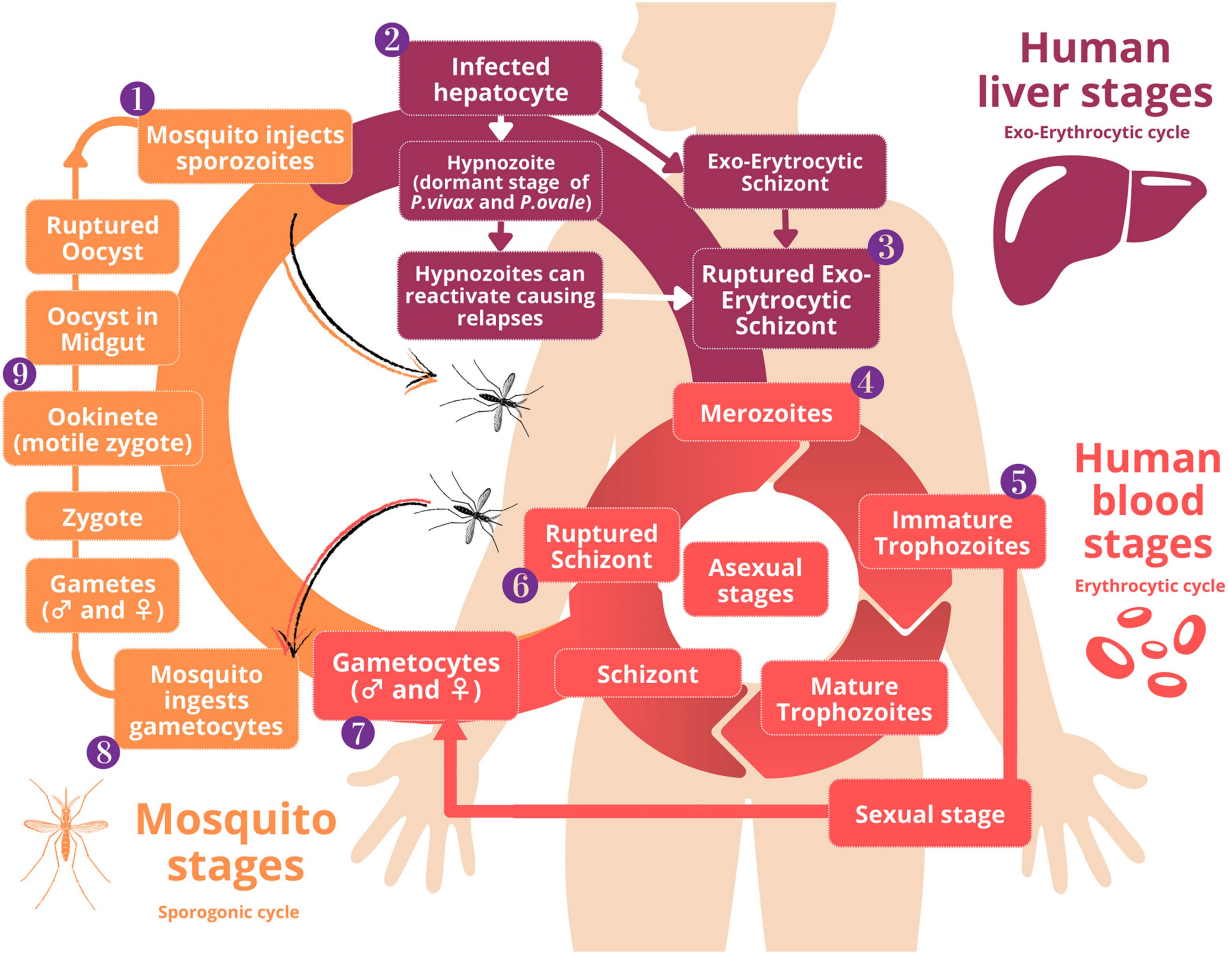
Life cycle of the *Plasmodium* parasite. Mosquito, human liver, and human blood stages are represented. **(1)** Mosquito injects sporozoites; **(2)** Infected hepatocyte; **(3)** Ruptured exo-erytrocytic schizont; **(4)** Merozoites in peripheral blood; **(5)** Immature trophozoites in peripheral blood; **(6)** Ruptured schizont; **(7)** Some immature trophozoites develop into sexual precursor cells named gametocytes; **(9)** Ookinete (motile zygote).

*Plasmodium* infection is produced by several protozoan parasites of the genus *Plasmodium* spp. (Tangpukdee et al., 2009). Five species of malaria cause infection in humans: *P. falciparum*, *P. vivax*, *P. ovale*, *P. malariae,* and *P. knowlesi*. *P. falciparum* is the most virulent species and produces the vast majority of deaths from severe malaria (Heide et al., 2019). The life cycle of *Plasmodium* parasites is represented in Figure 1. The life and infective cycle of the five species are similar, and their morphology and biology are analogous (Talapko et al., 2019).

Malaria treatment is crucial to reducing mortality. Prompt treatment is recommended, within 24 h of the onset of fever, and is fundamental for the reduction of mortality among children <5 years of age (Simba et al., 2018). After confirmation of *Plasmodium* infection by laboratory diagnostic techniques, such as Rapid Diagnostic Tests (RDT) or microscopy, anti-malarial drugs are administered. The treatment used should be determined by *Plasmodium* species, parasitaemia density, drug-resistant pattern where the infection was acquired, signs of severe malaria, and patient tolerance of oral medication (Griffith et al., 2007).

The implementation of early detection systems for malaria epidemics is a high priority in Sub-Saharan African regions (Guintran et al., 2006). New advances in the regulation and development of malaria vaccines, such as the RTS, S/ASO1 vaccine recommendation by the WHO, can reinvigorate the fight against malaria (*WHO recommends groundbreaking malaria vaccine for children at risk*, 2021). Laboratory techniques for malaria diagnosis by detecting *Plasmodium* parasites are extensively used worldwide; microscopic visualization of thin and thick blood smears is the gold standard technique for malaria diagnosis. RDTs are also used as recommended diagnostic tools and could be an affordable complement for a precise diagnosis due to their rapidness and easy handling. Both microscopic visualization and RDTs have their limitations and new diagnostic techniques are emerging to complement the tools used nowadays. As a breakthrough, new image analysis techniques based on deep learning, a subfield in artificial intelligence (AI), are being developed for the automated diagnosis of blood slides. Distinguishing between erythrocytes infected or uninfected with malaria parasites is possible with deep learning detection-based models. Image analysis techniques allow the detection of malaria parasites in digital images by pre-trained deep learning models with large image datasets. This process would emulate the optical microscope visualization of thick and thin blood smear samples and automate the procedure. Smartphone applications could integrate image analysis technology based on AI and would be an affordable option for resource-poor environments in endemic areas.

Identification of the different parasite morphologies in the whole *Plasmodium* life cycle is crucial to perform a correct diagnosis by microscopic examination of blood smears. The life cycle must be considered when experts perform manual labelling of digital images. Immature *P. falciparum* trophozoites (ring stage), White Blood Cells (WBCs), and erythrocytes are commonly labelled in malaria thick and thin blood smear digital images (Manescu et al., 2020). The labelled data would be used to train deep neural network models and create AI algorithms capable of detecting parasites and cells.

## Malaria diagnosis

Malaria diagnosis is crucial to treat and eradicate *Plasmodium* infections. An early diagnosis is determinant in effectively fighting against infection. Laboratory diagnosis is accepted worldwide and recommended for malaria detection (Tangpukdee et al., 2009; *World Malaria Report* WHO, 2021). Diagnostic methods for infectious diseases should be fast, accurate, simple, and affordable (Vila et al., 2017). Several techniques are available and used to directly or indirectly detect the presence of malaria parasites in blood. Table 1 shows the advantages and disadvantages of the most important diagnostic methods for malaria parasite detection.

**Table 1:** Advantages and disadvantages of malaria diagnostic techniques.

Diagnostic technique	Advantages	Disadvantages	References
Microscopic examination	(i) Availability (ii) Low-cost diagnosis (iii) Parasite level calculations (iv) Species identification	(i) Requires expert personnel (ii) Results are expert-dependent	Dowling and Shute (1966), Collins and Jeffery, (2007), Guintran et al. (2006), Wangai et al. (2011), Poostchi et al. (2018), Heide et al. (2019), *Malaria diagnosis and treatment* CDC, 2019
Quantitative Buffy Coat (QBC)	(i) Fast preparation and diagnosis results (ii) High sensitivity	(i) Requires expert personnel (ii) Requires fluorescent microscopy (iii) Specialized instrumentation	QBC Malaria Test, 2007, Tangpukdee et al. (2009), Siciliano and Alano (2015), *About Malaria* CDC, 2019, Shujatullah et al., 2006
Rapid Diagnostic Tests (RDTs)	(i) Fast preparation and diagnosis results (ii) Easy handling (iii) Low-cost diagnosis (iv) Species identification (usually *P. falciparum* from non-*P. falciparum* species)	(i) *pfHRP2/3* gene deletions (ii) Low sensitivity with low parasite levels (iii) Low sensitivity with *P. ovale* and *P. malariae* species. (iv) Cross-reactivity (v) Prozone effect	Wongsrichanalai et al. (2007), Gillet et al. (2009), Murray and Bennett (2009), Tangpukdee et al. (2009), Bejon et al. (2014), Nima et al. (2017), Orish et al. (2018), Cunningham et al. (2019), *Response plan to phrp2 gene deletions* (WHO, 2019), Ajakaye and Ibukunoluwa (2020), Kavanaugh et al. (2021), Kavanaugh et al. (2021)
PCR	(i) High sensitivity and specificity (ii) Species identification (iii) Reference tool for comparative studies	(i) Specialized instrumentation (ii) Difficult implementation in endemic areas (iii) Expensive diagnosis	Johnston et al. (2006), Li et al. (2014), Poostchi et al. (2018), Siwal et al. (2018), Haanshuus et al. (2019), Eshag et al. (2020), Leski et al. (2020), Feufack-Donfack et al. (2021), Mwenda et al. (2021)
LAMP	(i) High sensitivity and specificity (ii) Species identification (iii) No thermocyclers needed	(i) Specialized instrumentation (ii) Expensive diagnosis	Ocker et al. (2016), Selvarajah et al. (2020), Morris and Aydin-Schmidt (2021)
Serology	(i) Seroprevalence (ii) Malaria transmission	(i) Non-reliable diagnostic technique (ii) Not indicative of active infection	Tangpukdee et al. (2009), Oviedo et al. (2020)
Flow cytometry	(i) Quantification of infected erythrocytes (ii) Automated parasite level calculations	(i) Low sensitivity (ii) Specialized instrumentation (iii) Difficult implementation in endemic areas	Poostchi et al. (2018), Khartabil et al. (2022)
Biomarkers	(i) High diagnostic potential (ii) Easy handling	(i) Specialized instrumentation	Jain et al. (2014), Krampa et al. (2017))

QBC: Quantitative Buffy Coat, RDTs: Rapid Diagnostic Tests, PCR: Polymerase Chain Reaction, LAMP: Loop-Mediated Isothermal Amplification, Serology, Flow cytometry, and biomarkers.

### Clinical diagnosis

Clinical diagnosis is the least expensive option for malaria diagnosis (Wongsrichanalai et al., 2007), although the non-specific symptomatology and possible confusion with other infections or diseases with similar manifestations could overlap with the final diagnosis. Patient origin, malaria season, and age group are important aspects to consider. Clinical symptomatology could vary depending on the phase of the disease and the *Plasmodium* parasite species producing the infection. *Plasmodium* infection could produce asymptomatic, placental, uncomplicated, and severe malaria depending on the symptomatology and infection phase (Molyneux, 1989; Bartoloni and Zammarchi, 2012; Phillips et al., 2017).

Clinical symptomatology should be complemented with laboratory diagnostic techniques to confirm the presence of *Plasmodium* parasites. Blood smear samples are used in the vast majority of diagnostic techniques.

### Microscopic examination of blood smears

**Figure 2:**
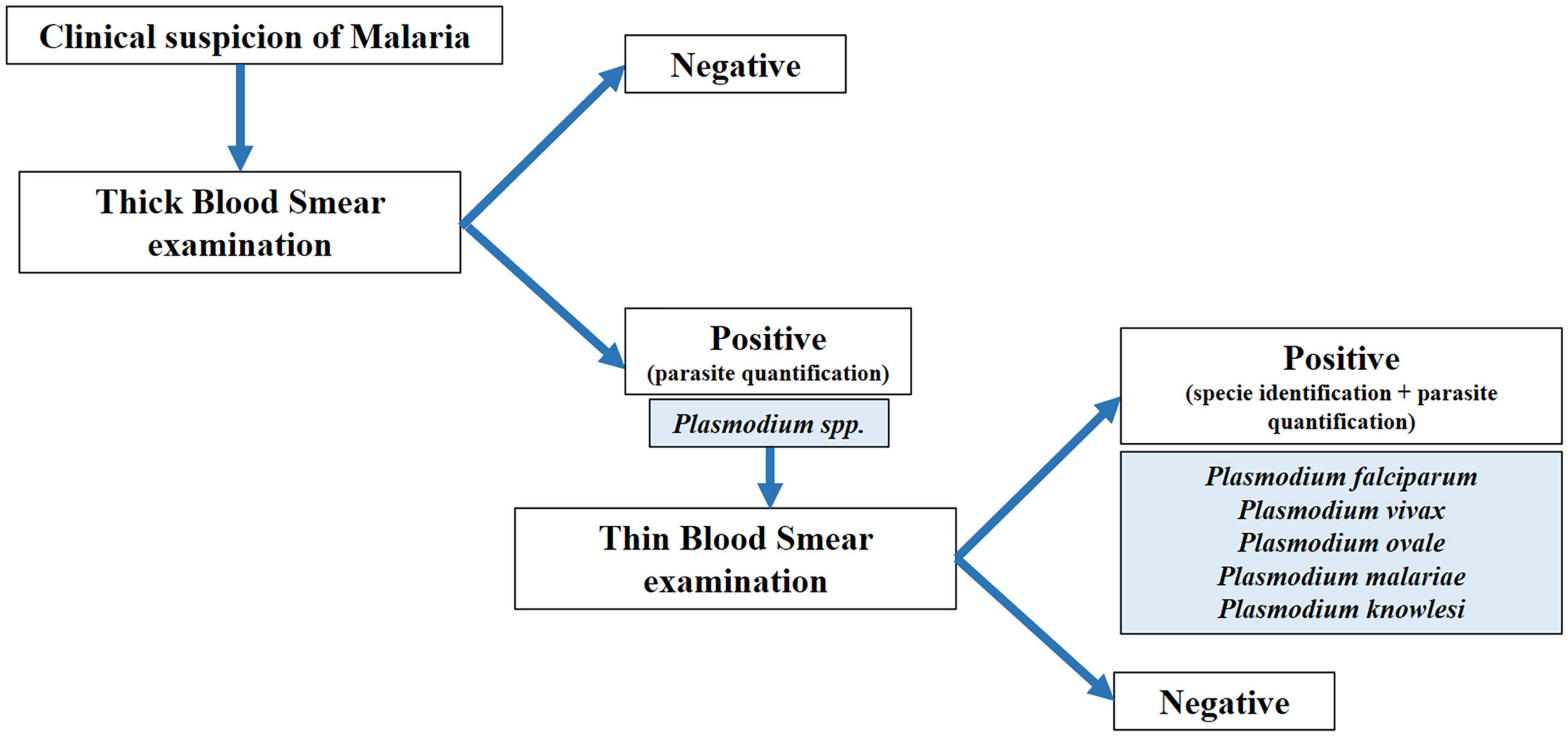
Blood smear microscopic examination procedure. Thick blood smear is first examined to determine the presence of malaria parasites. If the sample is positive, a thin blood smear is examined to determine *Plasmodium* species identification. Parasite quantification is performed to determine severity of the infection.

Direct microscopic examination of blood smears to observe malaria parasites is the gold standard technique for malaria diagnosis (Guintran et al., 2006; Collins and Jeffery, 2007; Heide et al., 2019). Prior to examination, the specimen is mostly stained with Giemsa or Leishman staining (Bejon et al., 2014), to afford the parasites a distinctive appearance (*Malaria diagnosis and treatment CDC*, 2019). The protocol for Giemsa staining of malaria blood films is a simple and fast technique to visualize the active form of parasites in blood (Turrientes and López, 2016). Malaria microscopy standard operating procedure is the protocol recommended by the WHO (*Giemsa staining of malaria blood films* WHO, 2016). The blood smear examination procedure is shown in Figure 2.

Knowing the life cycle of *Plasmodium* parasites (Figure 1) is important to perform a correct identification of the different developmental stages of the parasites and the species for diagnosis. *P. falciparum* usually causes higher parasite levels and produces most malaria deaths in Africa (*World Malaria Report* WHO, 2021). Maurer dots, poly-infected erythrocytes, and the characteristic banana shape of gametocytes are distinctive traits of *P. falciparum* infection (Zekar and Sharman, 2021). *P. vivax* and *P. ovale* are species sharing some similarities in the shape of parasites and quiescent liver forms. Both species infect young erythrocytes, have Schüffner’s dots, tend not to have multiple rings per cell, and contain malarial pigment. *P. malariae* usually causes lower parasite levels, due to its 72-h development cycle (24 h longer than *P. falciparum* and *P. vivax*), the lower production of merozoites per erythrocytic cycle, the predilection of parasites to develop inside old erythrocytes and the earlier development of immunity due to the combination of the previous factors (Collins and Jeffery, 2007). *P. knowlesi* is mostly present in Southeast Asia and was originally known as simian malaria. Due to its 24-h development asexual cycle, *P. knowlesi* infection can rapidly progress into severe malaria. Ring stage forms of *P. knowlesi* resemble *P. falciparum* and mature trophozoites and schizonts are similar to *P. malariae* forms (Amir et al., 2018). Gametocytes, the sexual stage of the parasite, are not responsible for clinical symptoms (*Treatment of malaria CDC*, 2013).

Microscopic visualization of thin blood smears allows the *Plasmodium* species identification from erythrocyte morphology and the distinctive features depending on the type of specimen infection. Thick blood smears are more efficient and provide higher sensitivity than thin blood smears (Wangai et al., 2011). The combination of both methods allows experts to determine the type and severity of the infection with a precise diagnosis. Parasite level calculations are performed manually in both types of samples. Direct microscopy observation is a tedious and time-consuming technique that requires experience and training. Continuous visualization of blood smears could trigger diagnostic errors due to the difficulty of the procedure (Dowling and Shute, 1966). The quality of the microscope and the staining reagents are also limiting factors (*Malaria diagnosis and treatment CDC*, 2019). False-negative cases lead to the unnecessary use of antibiotics, other consultations and, in some cases, progression to severe malaria. False-positive cases imply a misdiagnosis, unnecessary use of anti-malaria drugs, and suffering their potential side effects (Poostchi et al., 2018). However, microscopic examination of thin and thick blood smears is commonly used in endemic areas and resource-poor settings, due to its availability and easy handling. Other diagnostic techniques could complement and improve traditional microscopic examination and resolve its limitations.

### Quantitative Buffy Coat

The Quantitative Buffy Coat (QBC) test is a qualitative screening method for rapidly detecting the presence of malaria parasites in centrifuged capillary and venous blood (*QBC Malaria Test*, 2007). Blood is centrifuged in specially coated QBC tubes and visualized by optical fluorescence microscopy. The technique is based on a density gradient that separates the blood cells and allows the identification of parasitic forms by fluorescent microscopic observation of the capillary tube. The dye commonly used is acridine orange, which allows the identification of parasites between the erythrocyte and leukocyte areas. The QBC tubes also have an anticoagulant for the correct visualization of the sample and to avoid artefacts due to blood clotting (*QBC Malaria Test*, 2007). QBC presents higher sensitivity and specificity than conventional thick blood smear diagnosis due to the additional concentration of parasites in the narrow zone of the blood tubes (Siciliano and Alano, 2015; *About Malaria CDC*, 2019). This technique requires well-trained personnel, specialized instrumentation, is costlier than conventional light microscopy, and is difficult to determine the species and number of parasites (Tangpukdee et al., 2009).

### Rapid diagnostic tests

Rapid Diagnostic Tests (RDTs) are a suitable option and complement for detecting *Plasmodium* infection. RDTs are lateral-flow immunoassays that allow visualization of specific antigen–antibody recognition events (*Response plan to phrp2 gene deletions WHO*, 2019). They confer a qualitative diagnosis with a fast response time of less than 30 min (Cunningham et al., 2019). RDTs depend on the observation of a visible band on a nitrocellulose strip produced by the capture of dye-labelled antibodies. A drop of peripheral blood and a buffer solution are usually used to perform the diagnosis on the RDT device by detecting specific *Plasmodium* antigens. The majority of RDTs are based on the detection of the *P. falciparum*-specific protein histidine-rich protein II (HRP2) or universal antigen target for all malaria parasites, such as *Plasmodium* lactate dehydrogenase (p-LDH) or aldolase (Tangpukdee et al., 2009). HRP2 is localized in the cytoplasm of *P. falciparum* and on the surface membrane of infected erythrocytes (Murray and Bennett, 2009). Gene deletions of the parasite target gene *pfhrp2* are observed in several studies in endemic areas such as Ethiopia and Bangladesh (Wongsrichanalai et al., 2007; Bejon et al., 2014; *Giemsa staining of malaria blood films WHO*, 2016; *Treatment of malaria CDC*, 2013; Nima et al., 2017). False-negative results due to *pfhrp2/3* gene mutation could trigger an incorrect diagnosis. Low parasite density, incorrect interpretation of results, or *P. malariae* and *P. ovale* infections are also causes of false-negative results and reasons for an incorrect diagnosis by RDTs (Kavanaugh et al., 2021). A prozone effect due to excess antigen could trigger an incorrect diagnosis, although it is not a common event (Gillet et al., 2009). False-positive results are less common and can also trigger an incorrect diagnosis. Cross-reactivity due to high parasite levels or the presence of other disease antigens are the main causes of false-positive results (Orish et al., 2018; Kavanaugh et al., 2021; Pyle-Eilola et al., 2021). RDTs are a useful diagnostic support feature for conventional diagnosis, however, they cannot substitute microscopy examination (Ajakaye and Ibukunoluwa, 2020).

### Polymerase chain reaction

Polymerase Chain Reaction (PCR) diagnosis is a suitable alternative to conventional techniques. It is based on the amplification of *Plasmodium* DNA, and has high sensitivity, specificity and relatively low complexity (Leski et al., 2020). It is more sensitive than microscopy and capable of identifying malaria parasites at the species level when conventional methods are not able to detect the parasite (Johnston et al., 2006). In addition, the determination of *Plasmodium* species by PCR assay allows the unequivocal diagnosis in mixed species infection (Siwal et al., 2018) or low parasite levels (Haanshuus et al., 2019), which are difficult to detect by microscopic examination. Some of the main disadvantages of PCR diagnosis are the implementation of a non-routine technique in remote areas, the long-time (2–3 h) needed for diagnosis, and the high cost of the technology (Poostchi et al., 2018). Nowadays, PCR is being implemented as a diagnostic technique for malaria, although it is not the gold standard procedure and is not more widely used in endemic countries. Molecular techniques are useful to detect asymptomatic patients or those with very low parasite levels; their performance with this casuistry is considerably better than the other diagnostic techniques employed (Mwenda et al., 2021). This molecular diagnosis technique is commonly used in high-income countries or regions to perform epidemiological studies (Li et al., 2014; Eshag et al., 2020; Feufack-Donfack et al., 2021). As an example, novel PCR assay, such as MC004 RT-PCR, is demonstrated to be a useful tool for clinical settings and has a high degree of sensitivity and specificity (Beyene et al., 2022).

### Loop-mediated isothermal amplification

Loop-Mediated Isothermal Amplification (LAMP) is a molecular technique based on the amplification of nucleic acids employing *Bacillus stearothermophilus* DNA polymerase (Morris and Aydin-Schmidt, 2021). It has a 99% sensitivity and 93% specificity for malaria parasite detection compared with microscopy and does not require thermocyclers (Ocker et al., 2016). A fluorescence spectrophotometer is usually needed to read-out diagnostic results, which restricts the applicability in rural areas. However, new LAMP assays are designed with a fluorescence readout unit in order to detect *P. falciparum* parasites (Puri et al., 2022). It is not widely implemented as a diagnostic method, although it is postulated as an interesting alternative to conventional PCR methods and could be progressively implemented in resource-poor settings (Selvarajah et al., 2020).

### Other diagnostic techniques

**Serology** is based on the detection of antibodies against blood-stage malaria parasites. It is not commonly used for a rapid malaria diagnosis, although it is mainly used to perform seroprevalence studies of the disease. As an example, Immunofluorescence Antibody Testing (IFA) uses specific antigens for the quantification of IgG and IgM antibodies in serum samples (Tangpukdee et al., 2009). Combined strategies using serological, antigen detection, and DNA data are used to estimate malaria transmission and perform epidemiological studies (Oviedo et al., 2020).

**Flow cytometry** is a laser-based cell counting method that allows the quantification of erythrocytes infected by malaria parasites. It offers automated parasite level counts and has a low sensitivity (Poostchi et al., 2018). New advances based on fluorescence flow cytometry have shown that the Sysmex XN-31 device can determine the *Plasmodium* species and quantify parasites in blood. However, it can generate false positive results in case of abnormal erythrocytes cell morphology and the device was tested in a non-endemic region (Khartabil et al., 2022).

**Biomarkers** are cellular, biochemical, or molecular alterations that indicate the presence of biological, pathogenic, or therapeutic responses, with a high potential for diagnosis (Jain et al., 2014). The development of malaria biomarker detection, multiplex biomarkers for multiple *Plasmodium* parasite infections, and biosensors are new improvements to be considered as diagnostic tools (Krampa et al., 2017).

### Diagnostic methods comparison

To perform a comparison between the different diagnostic methods for malaria parasite detection it is important to consider the parasite levels. Low parasite levels are related to lower sensitivity values due to the less number of parasites in blood. Higher parasite levels are easier to detect with all the aforementioned techniques, although in some specific cases, a prozone effect could trigger antigen detection issues by RDTs (Gillet et al., 2009). The commercial brand of the techniques (QBC, RDTs, and PCR), RDT storage conditions, and response time are crucial for the correct interpretation of diagnostic results and could affect the final outcome. In the case of thick and thin blood smears, the expertise of the microscopist is determinant. The reference technique used as the standard against which others are compared to evaluate the quality of the method is also decisive (Feleke et al., 2021). Table 2 shows the diagnostic methods most commonly used for malaria parasite detection in terms of sensitivity and specificity. In some cases, no differentiation between thick and thin blood smear samples was observed in comparative studies and meta-analyses to determine sensitivity and specificity. However, thick blood smears provide a higher sensitivity than thin blood smear samples (Wangai et al., 2011). PCR is considered to have 100% sensitivity and specificity and is usually used as the reference method.

**Table 2:** Sensitivity and specificity of malaria diagnostic methods.

Diagnostic methods	Sensitivity	Specificity	Specifications	References
Microscopy	75.20%	97.12%	Comparative study.**Thick blood** films are 20–40 times more sensitive than **thin blood** films. Parasite density interferes with the final result and is crucial to obtain a reliable conclusion.	Bejon et al. (2006), Wangai et al. (2011), Feleke et al. (2021)
QBC	55.9%	88.8%	Lagos State University Teaching Hospital.	Adeoye and Nga (2007)
	70.5%	92.1%	University College Hospital, Ibadan, Oyo State, Nigeria.	Ifeorah et al. (2017)
RDTs	84.2%	99.8%	BinaxNOW test.	DiMaio et al. (2012)
	63.4–100%	53.4–99.9%	Mixed brands (Comparative study).	Boyce and O’Meara (2017)
	84.2%	95.2%	University College Hospital, Ibadan, Oyo State, Nigeria / pLDH RDT Optimal.	Ifeorah et al. (2017)
	37–88% (37 and 51% in asympt.)	93–100% (28% in one outlier)	Mixed brands (Comparative study).	Feleke et al. (2021)
LAMP	100%	86–99%	LAMP compared with PCR.	Feleke et al. (2021), Puri et al. (2022)
	95–98%	91–99%	LAMP compared with PCR.	Morris and Aydin-Schmidt (2021)
PCR	Considered 100%	Considered 100%	Used as a reference to be compared with other techniques.	Feleke et al. (2021), Mwenda et al. (2021)

QBC: Quantitative Buffy Coat, RDTs: Rapid Diagnostic Tests, pLDH: *Plasmodium* lactose dehydrogenase, LAMP: Loop-Mediated Isothermal Amplification, PCR: Polymerase Chain Reaction, asympt: Asymptomatic.

## Novel diagnostic tools by using image analysis techniques

The global health impact of malaria has accelerated the development and implementation of novel diagnostic strategies to fight against the disease. Novel diagnostic techniques based on image analysis and AI are being developed for malaria parasite detection; an emulation of microscopic visualization by image capturing and processing could be a fast and efficient alternative to performing the diagnosis. In the last years, computational microscopic imaging methods for object detection have held higher importance in medical and biomedical studies (Das et al., 2015). Several software applications and tools are being developed to detect malaria parasites in thick and thin blood smear sample images using conventional light microscopy (Luengo-Oroz et al., 2012; Dallet et al., 2014; Das et al., 2015; Pirnstill and Coté, 2015; Bashir et al., 2017; Oliveira et al., 2017; Laketa, 2018; Manescu et al., 2020; Yang et al., 2020; Yu et al., 2020).

Deep learning is a set of computational AI processes and methodologies that allow automated learning and the generation of algorithms by emulating the human brain. It is based on databases information, and uses artificial neural networks with multiple layers to train and generate AI algorithms (Alzubaidi et al., 2021). Deep learning has, in many aspects, boosted and improved the procedure for traditional computer vision imaging techniques (M K. Georgieff, 2016). Convolutional Neural Networks (CNN) are artificial neural networks widely used as trained classifier models to detect objects in images or videos by deep learning algorithms. Specifically, CNN classification is applied in medical diagnosis to analyse and extract efficient features from images as an AI healthcare tool (Sarvamangala and Kulkarni, 2021). Imaging radiology techniques for early diagnosis and treatment of emerging infectious diseases such as Zika, Ebola, or Chikungunya are other image analysis applications (Jardon et al., 2019). Microscope image analysis using a U-Net (convolutional network architecture) to segment and detect Leishmaniosis (Górriz et al., 2018) is a representative study of the wide variety of possibilities of CNNs. The high computing capacity achieved over the past years and the increased amount of training data for CNNs have boosted the use of this technology for medical applications (O’Mahony et al., 2020).

In particular, automated microscopy imaging analysis could also be an alternative to conventional microscopy examination for malaria diagnosis. The preparation and type of sample are important facts to consider to perform the correct identification of biological features. Table 3 summarizes the visual image differences between thick and thin blood smears and their analysis by AI techniques (Ross et al., 2006; Mushabe et al., 2013; Dallet et al., 2014; Oliveira et al., 2017; Sankaran et al., 2017; Dantas Oliveira et al., 2018). Thick blood smear examination is crucial for a correct diagnosis of malaria, allowing the consequent visualization of thin blood smears for species identification (Figure 2). Thick blood smears are more sensitive and appropriate for low malaria parasite levels (Dowling and Shute, 1966). Nevertheless, the frequency of artefacts observed in this type of sample is higher in comparison with thin blood smears (Prairie, 2012).

**Table 3:** Visual image differences between thick and thin blood smear samples to distinguish malaria forms by artificial intelligence techniques. **(A)** Thick blood smear sample 1,000x Giemsa staining. WBC nuclei and immature trophozoites (T) are distinguished with an arrow. **(B)** Thin blood smear sample 1,000x Giemsa staining. Erythrocytes infected with young trophozoites (T) of *P. falciparum* and uninfected erythrocytes (RBC) distinguished with an arrow. Maurer dots are present in infected erythrocyte morphology.

Thick blood smear sample	Thin blood smear sample
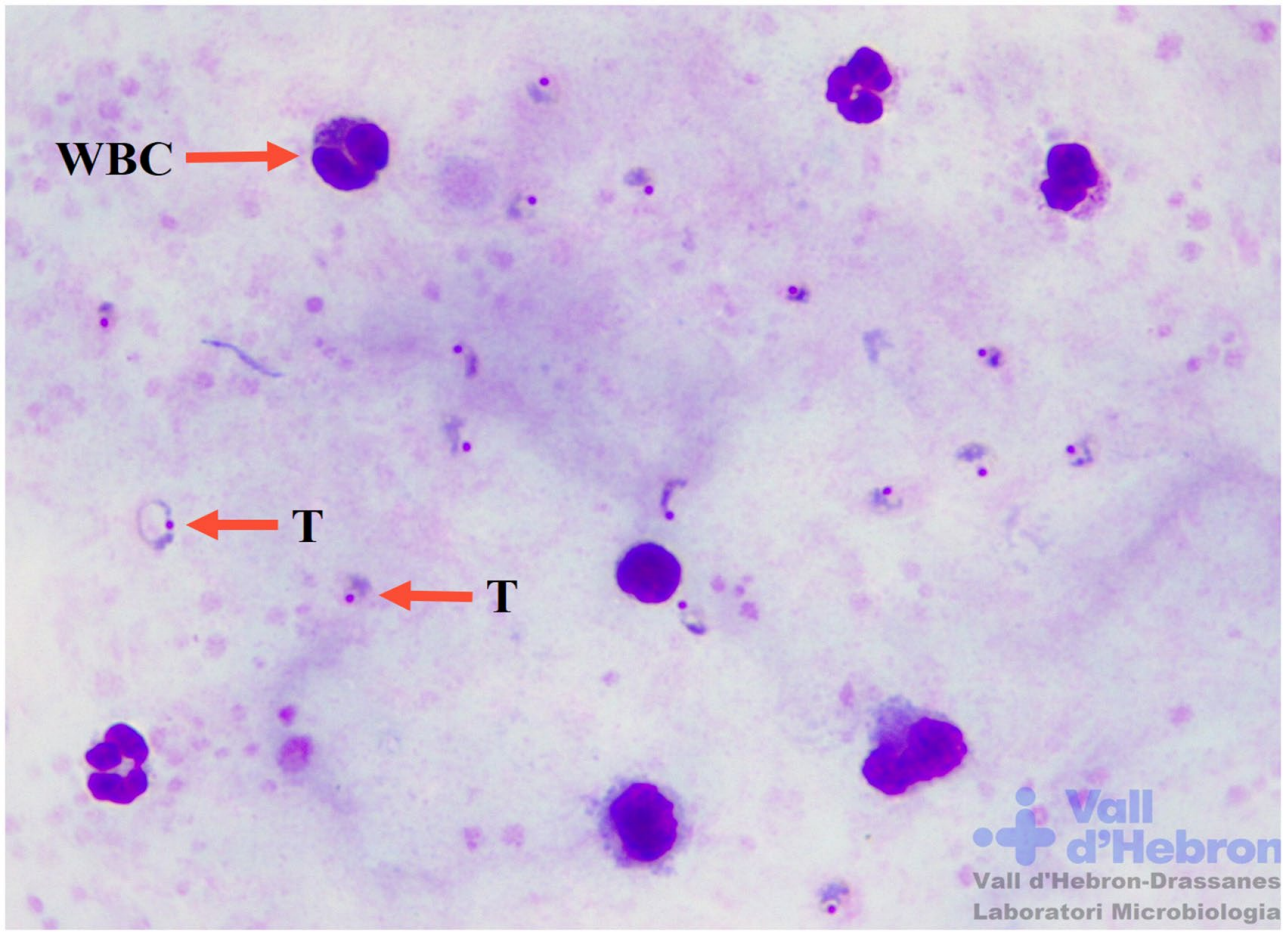 (A)	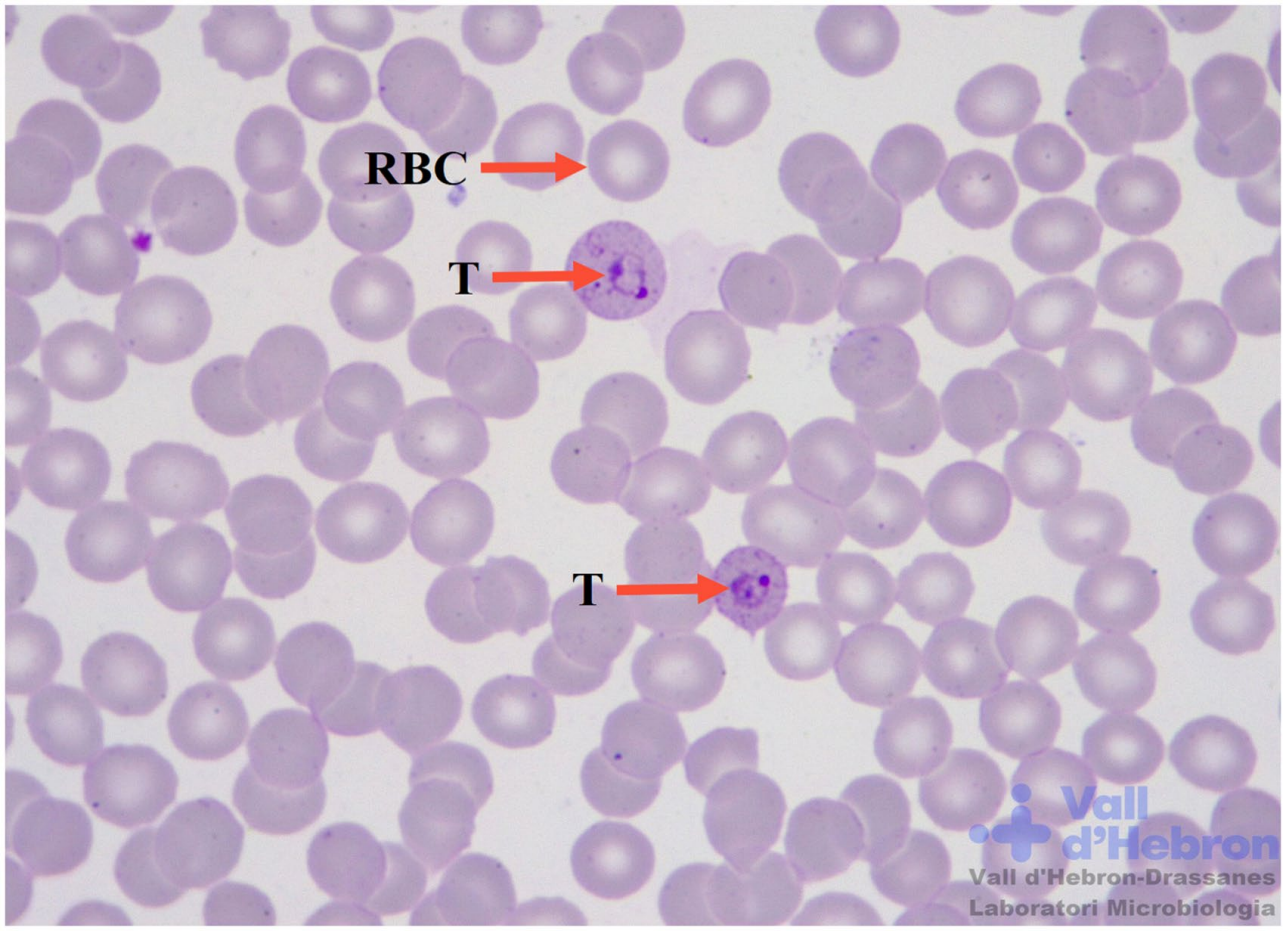 (B)
Main features	Main features
– Positive/Negative diagnosis. – Possible to distinguish all development stages of the blood life cycle of the parasites. – Non-species identification (except in the case of *P. falciparum* gametocytes). – Haemolysis of erythrocyte cells. – Amorphous morphology of immature *Plasmodium* trophozoite cytoplasm. – High sensitivity. – Common appearance of Giemsa artefacts.	– *Plasmodium* species identification by parasite and erythrocyte morphology. – Parasite development stages identification inside erythrocytes. – High specificity. – Fewer artefacts and confusion forms. – Fixing sample with methanol in Giemsa staining technique. – Erythrocyte and staining artefacts.
– Thick blood smear malaria parasite detection by artificial intelligence imaging tools (Rosado et al., 2016; Xiong et al., 2019; Manescu et al., 2020; Yang et al., 2020; Yu et al., 2020).	– Thin blood smear malaria parasite detection by artificial intelligence imaging tools (Ross et al., 2006; Tek et al., 2010; Dallet et al., 2014; Kareem et al., 2012; Mushabe et al., 2013; Oliveira et al., 2017; Rosado et al., 2017; Sankaran et al., 2017; Dantas Oliveira et al., 2018; Pillay et al., 2019; Pardede et al., 2020; Yu et al., 2020; Abubakar et al., 2021; Davidson et al., 2021).

CNNs for the detection of malaria parasites in thick blood smears are less used in comparison with thin blood smears. New automated parasite detection in thick blood smears based on deep learning and neural networks is an optimal alternative to traditional parasite microscopy visualization, as demonstrated in several studies (Xiong et al., 2019; Manescu et al., 2020; Yang et al., 2020). Other important factors for the visualization of thick blood smears are erythrocyte haemolysis, WBC cytoplasm rupture, and the variable shapes of ring-stage trophozoites. Most of the methods published for malaria parasite identification are based on supervised procedures that require a previous manual labelling procedure of microscopic images. Malaria digital images of thick and thin blood smears need to be labelled to create a dataset large enough to allow the generation of an optimal detection model (Shambhu et al., 2022). This process requires to manually define the bounding box of each parasite of a set of images to train the neural network model.

### Image acquisition

Image capturing/acquisition is the first step towards generating an image database for future analysis and identification. Acquisition depends on the equipment and infrastructure of the laboratories. Microscope-integrated cameras are often used to acquire digital images with conventional light microscopy. However, smartphone cameras with an adapter bracket are an affordable alternative for automated malaria diagnosis applications (Srikanth et al., 2008; Dallet et al., 2014; Rosado et al., 2016, 2017; Oliveira et al., 2017; Yu et al., 2020). Thus, in low-income countries, smartphone cameras would be a useful tool for acquiring digital images and replacing integrated microscope cameras, which are usually more expensive. The quality and resolution of the digital image, pixel morphology and density would determine future image processing and analysis. Other types of techniques for acquiring malaria parasite images with different microscopes are also used, such as fluorescent microscopy, binocular microscopy, or polarized microscopy (Poostchi et al., 2018). Nevertheless, image acquisition with conventional light microscopy is the most similar procedure to emulate conventional microscopic malaria diagnosis in endemic countries. Image acquisition is the first step for both traditional image processing techniques and deep learning methods (Hegde et al., 2019).

### Traditional image processing techniques for malaria parasite detection

Image pre-processing is used in traditional computer vision techniques to automatically detect parasites and allows the preparation of acquired images to improve further analysis. Most studies perform noise reduction, enhancement of image contrast, and image resizing. These modifications would facilitate future procedures of feature extraction. As an example, Gaussian average filters or low-pass filters are used to reduce the noise of malaria microscopy images (Fatima and Farid, 2020). Moreover, background image assumption and colour normalization and correction to reduce the effects of illumination is an affordable solution to reduce image errors (Tek et al., 2016). Colour normalization and grey world-based colour normalization are pre-processing methods to minimise sample staining issues that could trigger image artefacts. Pre-processing imaging methods for smartphone image acquisition by colour normalization and background removal are useful tools to prepare images for the automated diagnosis of leishmaniasis or bartonellosis in remote locations (Cesario et al., 2012). Image resolution and quality are decisive to perform a correct and precise diagnosis *via* imaging methods.

Image segmentation is very often required to extract features. Segmentation consists of classifying each pixel as part of the objects in the original image. Morphological operations, Hough transform, K-means clustering, watershed algorithm, edge-based segmentation algorithms, rule-based segmentation, template matching, and marker-controlled watershed are segmentation techniques used for thin and thick blood smear images, among other applications (Poostchi et al., 2018). Many of these are complemented with thresholding techniques as a final step to extract and define the different segmented regions.

Feature extraction is the next procedure. The characterization of thin and thick blood smear images by features such as staining colours, cell texture, and morphology are carefully chosen (Poostchi et al., 2018). For example, erythrocyte feature calculations in thin blood smear images are performed by open-source platforms such as PyRadiomics 2.2.0 (Savkare and Narote, 2015). Feature extraction facilitates the subsequent learning and classification steps by providing quantitative information on certain image parameters.

Machine learning or pattern recognition is the final step of the image analysis procedure before identification. Classification methods are used for the identification of parasites and WBCs in thick blood smear samples, or infected and uninfected erythrocytes in thin blood smears. It is important to distinguish between the parasite identification procedures for the two sample types. In both cases, the performance of the technology developed should be optimized in terms of accuracy, sensitivity, and specificity (Poostchi et al., 2018). Most articles published on the identification of malaria parasites in thick blood smears are for *P. falciparum* infections (Yang et al., 2020). Thin blood smear parasite identification is used to distinguish between erythrocytes infected or uninfected with malaria parasites. In addition, parasite species identification and the development stage of the parasite in thin blood smears are detected by using traditional pattern recognition techniques that include, for example, Support Vector Machine (SVM) or logistic regression classifiers (Tek et al., 2010). Response time depends on the computational complexity of the predictive model. Complexity increases the time of response, although an evaluation between complexity and time is crucial to perform a correct and sufficiently fast identification (Freire et al., 2021).

### Convolutional neural networks for malaria parasite detection

Convolutional neural networks are computational systems inspired by biological neurons designed to process data (Anwar et al., 2018). Image input is analysed to recognize visual patterns and complete the future identification of objects as an output. Neurons in deep networks are controlled by an activation function, which is responsible for controlling the output. Operations such as pooling and regularizers, with L1, L2 norms, batch normalization, or dropout are key elements to make the predictive models learn better and faster (Goodfellow et al., 2016; Anwar et al., 2018). Overfitting issue due to a memorization of data instead of learning could interfere in the final training outcome and obtaining of robust final predictions (Demšar and Zupan, 2021).

An important fact to consider when training CNNs is to have sufficient representative data. Data is commonly distributed into three sets: training, validation, and testing. The prediction model learns from the multiple examples of the dataset and the same training data is fed into the CNN repeatedly in an iterative procedure. During training, the validation dataset allows hyperparameter tuning and model evaluation by a continuous optimization. Finally, a test dataset is used to assess the model after completing the training process with unseen data (Xu and Goodacre, 2018).

Object detection deep learning models are able to identify and locate objects of a certain class in images and videos (Jiao et al., 2019). During the last few years, object detection models have been improved and most of the state-of-the-art object detectors use deep learning networks. Usually, raw images need a simple pre-processing to resize them and fed them into the network. The model itself decides and computes the appropriate features and provides an output that leads to the identification and location of objects. Among other uses, medical imaging may benefit from object detection techniques, in particular, it could be a useful alternative to malaria parasite detection (Jiao et al., 2019).

Object detectors are classified as two-stage or one-stage. Two-stage detectors have high localization and object recognition accuracy, whereas one-stage detectors achieve high inference speed (Jiao et al., 2019). The most representative two-stage detector is Faster R-CNN (Ren et al., 2017) and one-stage object characteristic detectors are YOLO (Redmon et al., 2016) and SSD (Konishi et al., 2016).

In most cases, manually labelled data is required to perform all the aforementioned processes. Unsupervised training is an alternative, although most medical imaging studies are performed with supervised training data. Supervised learning based on image annotation is diverse and several strategies have been described (Sarangi, 2014). Whole-image classification is the annotation of the whole image as a type or class. Non-discerning objects are detected in the image, so the whole image is classified as a type. Object detection using bounding boxes within each image is another option when solving classification tasks. It requires a more time-consuming supervised annotation procedure of the different objects in the image. CNNs use the dataset and identify every bounding box as an object class (Ibrahem et al., 2022).

In the case of image segmentation, the identification of objects is based on a pixel-by-level classification. Each pixel is classified as a class object with its own value and annotations are manually added to images. However, it is an even higher time-consuming task for large databases, therefore automatic annotation procedures are being developed. Thus, other conventional machine learning methods and deep learning procedures are used to automatically annotate images (Murthy et al., 2015; Cao et al., 2020).

Furthermore, CNNs need large datasets with annotated data. ImageNet is one of the largest available datasets of universal images for researchers and non-commercial use (ImageNet, 2021). In the particular case of malaria, a sufficiently large dataset of malaria annotated images is needed to train CNN models and perform an automated identification of parasites. Malaria thick blood smears from the Institute of Electrical and Electronics Engineers (IEEE) DataPort is an open-source image dataset (*Malaria Thick Blood Smears | IEEE DataPort*, 2021). Strikingly, there are not many publicly available datasets of malaria thick and thin blood smear images. Data augmentation techniques, to artificially enlarge image datasets and obtain better performances, is nowadays used with promising results as DACNN model demonstrates (Oyewola et al., 2022).

**Figure 3:**
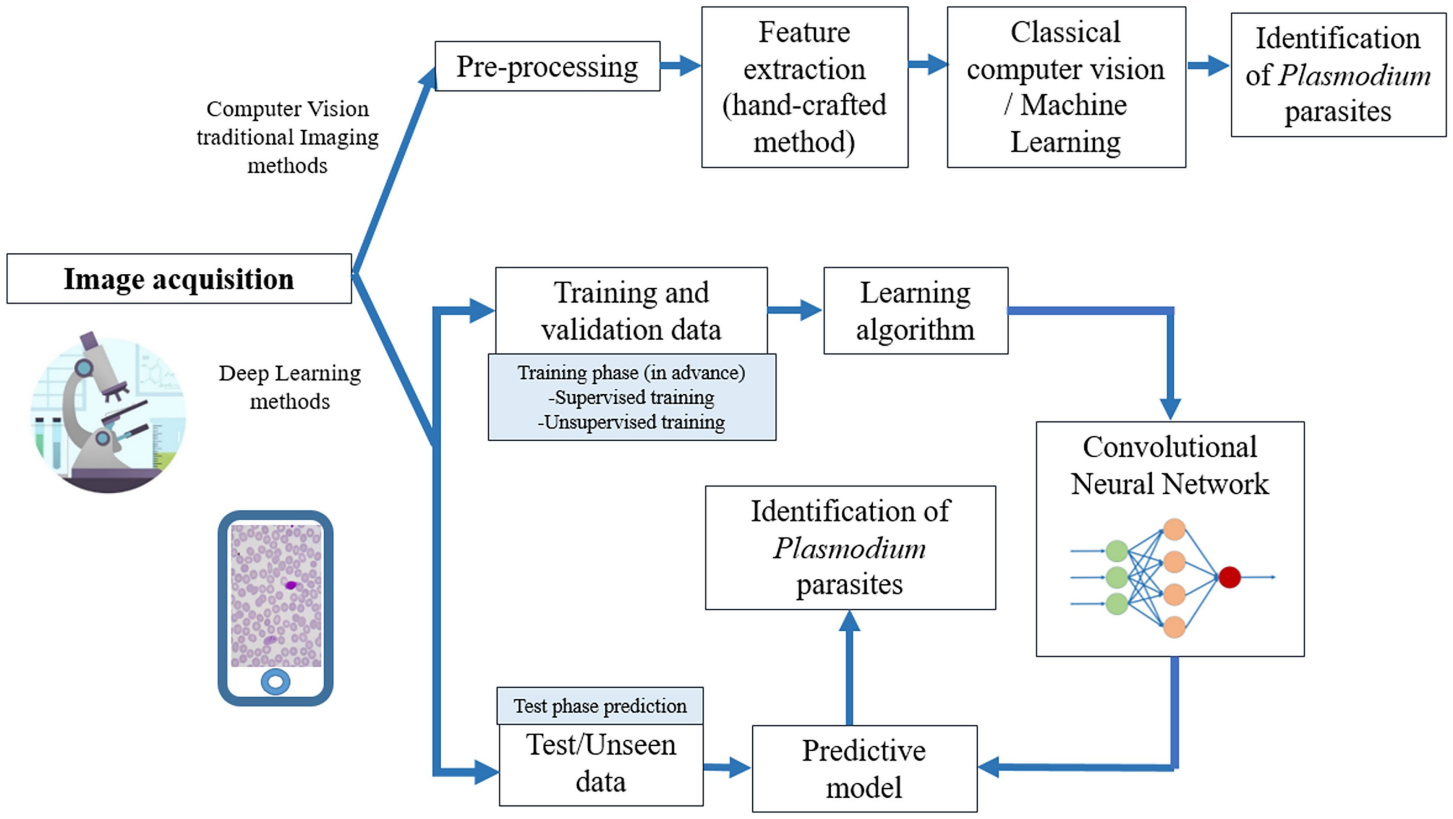
Representation of the different procedures using Traditional Computer Vision Imaging methods or Deep Learning methods (Convolutional Neural Networks) for malaria parasite identification in thick and thin blood smear samples.

CNNs have been shown to have optimum performance with computer-aided image diagnosis applications in specific fields of study and can be generalised for other medical imaging tasks (Shin et al., 2016). Object detection models, such as YOLOv3, YOLOv4 and YOLOv5 are used for malaria parasite detection (Abdurahman et al., 2021; Rocha et al., 2022). Feature scale and addition of detection layers are modifications that provide better performances than state-of-the-art articles. Moreover, Faster R-CNN (Hung and Carpenter, 2017; Ren et al., 2017) and SPPnet (Zhou et al., 2018) are optimized neural networks used to speed up and enhance identification time. Recent studies demonstrate the potential of CNNs for malaria parasite detection with promising results, such as VGG-19 model by transfer learning mechanism (Alnussairi and İbrahim, 2022; Jameela et al., 2022) or transformer-based models to obtain optimized performance parameters (Islam et al., 2022). The general procedure for malaria parasite detection using deep learning imaging methods is represented in the bottom part of Figure 3. Nowadays, CNNs have improved and replaced the use of traditional methods.

### Automated malaria parasite level calculations using deep learning methods

Automated parasitaemia counting by image analysis is a useful tool that could overcome and provide support to manual parasite level quantification. Conventional malaria parasite level calculations by microscopy visualization of thick and thin blood smears are not precise and difficult to reproduce. An estimation is usually performed and, when parasite levels are high, is tedious and time-consuming. Thick blood smear parasite level quantification is routinely performed by counting the number of parasites and leukocytes in a blood smear sample (WHO and Regional Office for the Western Pacific, 2016). Thin blood smear quantification is based on the counting of infected erythrocyte cells in each microscopy field (WHO and Regional Office for the Western Pacific, 2016). Quantification of parasite levels *via* digital image analysis techniques would require the shortest period of time. An image analysis software was developed to perform this function automatically with thick blood smear images (Arco et al., 2015). For thin blood smear automated parasitaemia calculations there are image analysis tools available to improve conventional manual counting. Determination of malaria *P. vivax* parasite concentration is possible using image processing techniques (Prasad et al., 2020). *Plasmodium* AutoCount is a digital image analysis tool to perform an automatic count of parasites in Giemsa-stained thin blood smears (Ma et al., 2010). Other image processing tools based on OpenCV software libraries were satisfactory in determining parasite levels in thin blood smear samples (Swain et al., 2018). Previously mentioned methods used image processing techniques, such as noise reduction with filters and binary transformations, to determine the presence of malaria parasites inside erythrocytes and perform a final parasite level calculation.

### Mobile phone applications for malaria parasite detection

Mobile phone applications are being developed for the automatic detection of malaria parasites (Cesario et al., 2012; Rosado et al., 2016, 2017; Oliveira et al., 2017; Yu et al., 2020; Zhao et al., 2020). Smartphone image capturing is a suitable and easy alternative for the acquisition of blood smear images through the microscope lens. Only an optical microscope, a mobile adapter, and a conventional smartphone are needed to perform an imaging diagnosis. Mobile phone cameras could substitute integrated or external microscopy cameras and perform an optimum diagnosis by image analysis.

The integration of CNN predictive models in a smartphone software application is possible. Adapted CNN models perform the entire diagnosis in a single device. The coalescence between malaria automated diagnosis and smartphone software is a milestone and challenge for future implementation in worldwide laboratories. Image analysis and deep learning procedures allow smartphones to be one of the best alternatives for the implementation of automated malaria parasite detection. Even in resource-poor settings, smartphones are an available and relatively cheap option. One of the main problems of smartphone cameras was image quality and adaptation to microscopy lenses. Nowadays, smartphone cameras provide high image quality, although adaptation to the microscope is not as good as expected. Images could be disturbed by light microscopy issues, lens adaptation to smartphone cameras, or image quality downgrades related to image focus. Microscope image auto-focus is also an issue to solve. The technology to fully automate the entire procedure of image focusing, image acquisition, and parasite identification by an independent device is still required.

As an example, Malaria Screener is an affordable and effective solution for automatic malaria parasite detection by a mobile phone application (Yu et al., 2020). It combines image acquisition, smear image analysis, and result visualization. It is a semi-automated system based on digital images and CNN models to predict the presence of malaria *P. falciparum* parasites in thin and thick blood smears. Other applications were developed to combine automatic detection of malaria parasites *via* an optical magnification prototype with a smartphone device that performs image processing and analysis (Rosado et al., 2016). VGG16 classification CNN, or other CNN models, were integrated into smartphone applications to automatically detect the presence of malaria parasites inside erythrocytes in thin blood smear samples (Zhao et al., 2020).

Gamification of the technology for the identification of malaria parasites in digital images is also an innovative application. As an example, a web-based game where online volunteers analyse thick blood smear images to detect malaria parasites was developed for the creation of an annotated image database (Luengo-Oroz et al., 2012).

To sum up, smartphone applications might be the future for autonomous image acquisition and analysis by AI technologies, and a suitable alternative for malaria and Neglected Tropical Disease (NTDs) diagnosis. The possibility to integrate predictive models and image acquisition in a single device confers a wide range of applications in the field of image analysis for diagnostics.

### Microscopy automation linked to smartphone software technology

Microscopy automation to move blood smear samples and capture focused images automatically is a challenging approach. Automation would solve the limitations related to the non-fully autonomous diagnosis procedure performed. Image processing methods allow automation of the diagnosis, although a person is still needed to move the X-Y axis and issue focus of the microscope. A few studies have implemented automatic hardware devices to solve this problem and optimise the automation of malaria diagnosis (Kaewkamnerd et al., 2012; Gopakumar et al., 2018; Muthumbi et al., 2019). Microscopy adaptation is crucial to fully implement the aforementioned technology in real clinical and diagnosis practices. Low-cost hardware optimization with 3D printing models to manufacture specific parts or pieces of the microscope would be a suitable option in resource-poor settings with endemic malaria. A 3D-Printed portable robotic mobile-based microscope for the diagnosis of global health diseases is an example of the potential of this technology (García-Villena et al., 2021). As mentioned before, some studies present the possibility of developing an optical device that emulates or substitutes an optical microscope. An optical prototype with 1,000x magnification adapts to the smartphone camera and avoids possible light issues (Rosado et al., 2016). Nevertheless, conventional optical microscopy adaptation is the most suitable technique for image acquisition and analysis by smartphone applications.

### Implementation of malaria digital microscope imaging diagnosis in resource-poor settings

More than 90% of severe malaria produced by *P. falciparum* is estimated to affect young children under 5 years old in Sub-Saharan Africa, in areas with resource-poor settings (Schumacher and Spinelli, 2012; Gitta and Kilian, 2020). The gold standard method for malaria diagnosis by the WHO is still microscopy, although this is dependent on laboratory resources and could result in diagnostic errors due to a lack of instrumentation, medical devices, or well-trained laboratory staff. Microscopic examination of blood smears and RDTs are the techniques most used for malaria diagnosis and improvements aimed at the development of new and better diagnostic techniques are being implemented in endemic areas (Gitta and Kilian, 2020). The increase of RDT usage in malaria-endemic areas is replacing microscopic examination of blood smears due to the lack of resources and well-trained personnel. In addition, the biosocial situation of mothers and children in resource-poor regions, such as Imo State in south-eastern Nigeria, has an impact on the increased appearance of complicated malaria cases (Iloh et al., 2013). A non-precise diagnosis or treatment due to the low availability of resources is a serious issue in endemic areas. Consequently, the implementation of new and affordable diagnostic imaging techniques could help solve this problem.

Smartphones are a portable and suitable alternative for malaria diagnosis *via* imaging techniques, which could be implemented in resource-poor settings and remote endemic areas. They could improve and automate malaria diagnosis with less need for resources and personnel. CNN models could be integrated inside smartphone software and an internet connection would not be required. The provision of health centres with mobile devices by governmental organisations and national programs against malaria would be a determinant factor for the correct implementation of this novel technology for malaria diagnosis. However, due to the constraints specific to many malaria endemic areas, this may be a major problem to be addressed in the coming years by political willingness. The benefits of smartphones for diagnostics can be of significant value, not only for malaria, but also for the diagnosis of many other tropical diseases or NTDs (Vasiman et al., 2019). Due to that, implementation in regional hospitals or small healthcare centres would be a challenge for future studies. New object detection models trained with smartphone camera images are suitable for malaria diagnosis deployment in resource-poor settings (Abdurahman et al., 2021).

Diagnostic performance studies to validate the technology are a must for the future implementation of a tool. The performance evaluation should be carried out under ideal and resource-poor conditions to determine its effectiveness in different environments. There are many barriers to overcome in order to transition a product or technology from development to introduction and implementation. Some of the main barriers are the adjustment to the health and laboratory systems necessary to ensure effective adoption and implementation, demonstration of the technology’s value, evaluation of operational viability, policy and regulatory requirements of government organizations, operation research to evaluate the net effect of the technology in the field, distribution, service and repair, and quality assurance and control (Palamountain et al., 2012).

The implementation of new diagnostic techniques in laboratory environments has to be regulated and controlled by the Food and Drug Administration and WHO protocols (Palamountain et al., 2012). The technology should be validated and accepted by international and national authorities as described (Mugambi et al., 2018). Most efforts to implement new diagnostic tools in resource-poor settings are focused on infectious diseases such as HIV, Tuberculosis, and Malaria. Deep understanding and coordination of the stakeholders involved in the diagnostic development and implementation are milestones for the success of diagnostic interventions (Mugambi et al., 2018).

## Discussion and concluding remarks

Epidemic malaria is very prevalent in Sub-Saharan Africa and tropical regions with low resources. It is still a global health issue that should be solved by mosquito control strategies, rapid and accurate diagnosis, and correct treatment (*World Malaria Report* WHO, 2021). Therefore, diagnosis is crucial for the eradication of the disease and to reduce mortality in prevalent regions. However, the recurrent problems in these environments with conventional microscopic examination due to lack of resources and experimented microscopists (Ngasala and Bushukatale, 2019), and the increasing failure of RDTs mainly due to gene mutations (Golassa et al., 2020), reinforces the necessity of developing new, affordable, and accessible diagnostic methods for *Plasmodium* infection.

Advances in image analysis and processing allow and postulate the implementation of automated malaria diagnosis as a new diagnostic tool. Thick and thin blood smears would be the samples analysed by the new technology. Traditional image analysis techniques were used to automatically detect malaria parasites in thin and thick blood smears (Turrientes and López, 2016), as demonstrated in several studies (Tek et al., 2010). The irruption of deep learning methodologies with CNNs has boosted and improved the results for the identification of malaria parasites in comparison with traditional computer vision techniques. For CNN models and, specifically medical image processing and analysis, it is crucial to have a large image dataset to obtain reliable results. Unfortunately, open-source image datasets are not globally available and are usually used for individual CNN training.

Open image availability would be a beneficial resource for the scientific community. The ImageNet (ImageNet, 2021), parasite image (Li and Zhang, 2020), and malaria thick blood smear (*Malaria Thick Blood Smears | IEEE DataPort*, 2021) databases are representative examples. Neural networks such as YOLO are used as CNN models to detect malaria parasites in blood smears (Abdurahman et al., 2021).

In addition, the integration of CNN models into smartphone software is possible. Thus, the implementation of digital image analysis-based diagnostic tools in endemic areas with smartphone applications could improve and automate malaria diagnosis by the emulation of the gold standard microscopy examination technique. As an example, fully automated systems, such as the slide screening microscope EasyScan GO, evaluate their performance against WHO slide samples with promising results (Horning et al., 2021). CNN-based models are widely used as predictive models with the capacity to distinguish parasite forms and blood cells and could be implemented in low-resource settings (Zhao et al., 2020). Automated parasite detection, parasite level calculations, and faster diagnosis are some of the main advantages of image analysis for malaria diagnosis. This technology could be used as a fast and precise tool to perform parasite level calculations (Ma et al., 2010). Overall, the use of smartphones and artificial intelligence techniques for diagnosis might help the global goal of malaria eradication in the coming years. The support and enhancement of traditional microscopy-based diagnostic techniques through the use of AI, the upgrading of laboratory infrastructures in malaria endemic areas and the improvement of computer technology over the years may help to implement such techniques in most remote areas. Integrating innovations into the current microscopy method would reinforce malaria elimination (Nema et al., 2022).

Hardware automation is still in the process of optimization to complete the goal of independent predictive and mechanized diagnostics. Other limitations such as image quality dependence, laboratory infrastructure requirement, local regulatory organization permissions, or the necessity to create a standardized protocol for the final diagnosis should be addressed. Nevertheless, several studies are improving predictive models, pre-processing techniques, microscope automation, and faster detections (Sriporn et al., 2020; Masud et al., 2020). Artificial intelligence improvements and better predictive algorithms due to computing power evolution could be an advance in terms of automatic image diagnosis with optimized predictive results in the following years. In conclusion, with diagnostic techniques based on image analysis, the samples used are the same (thick and thin blood smear) and the procedure of sample preparation, parasite observation and interpretation would be very similar to conventional microscopy. In addition, it would provide technical support to health professionals and help to automate the process in order to increase its efficiency.

In this review, we have summarised the main advances, challenges, and limitations in the automation of malaria diagnosis using digital image analysis by AI tools. Smartphone applications are a suitable option to integrate diagnosis technology into a single device and confer laboratories a new tool for malaria and other disease diagnoses. New advances and improvements in AI would be the final milestone for the optimisation and implementation of the technology worldwide. In conclusion, we are ever closer to developing a fast, efficient, and optimum new diagnosis tool for malaria parasite detection available for laboratories located in malaria-endemic regions worldwide.

## Author contributions

CRM, ES, and JJ-M: conceptualized and drafted the manuscript, wrote the manuscript and designed the figures, AdO provided advice about artificial intelligence topics and designed the figures. FZ and ES provided continuous intellectual feedback about malaria diagnosis and the protocols used nowadays. JJ-M, ES, FZ, ES, DL-C, SN, BB, MB, AV, ME, AA, and TP revised the manuscript and provided valuable feedback. ES and JJ-M edited and revised the overall manuscript. All authors agreed to be accountable for the content of the work.

## Funding

The project is funded by the Microbiology Department of Vall d’Hebron Universitary Hospital, the Cooperation Centre of the Universitat Politècnica de Catalunya (CCD-UPC) and the Probitas Foundation.

## Conflict of interest

The authors declare that the research was conducted in the absence of any commercial or financial relationships that could be construed as a potential conflict of interest.

## Publisher’s note

All claims expressed in this article are solely those of the authors and do not necessarily represent those of their affiliated organizations, or those of the publisher, the editors and the reviewers. Any product that may be evaluated in this article, or claim that may be made by its manufacturer, is not guaranteed or endorsed by the publisher.
